# The Importance of Exclusion of Obstructive Sleep Apnea During Screening for Adrenal Adenoma and Diagnosis of Pheochromocytoma

**DOI:** 10.1177/2324709615607062

**Published:** 2015-09-19

**Authors:** Alicia C. Weeks, Michelle E. Kimple, Dawn Belt Davis

**Affiliations:** 1 University of Wisconsin, Madison, WI, USA; 2 William S. Middleton Memorial Veterans Hospital, Madison, WI, USA

**Keywords:** adrenal adenoma, pheochromocytoma, obstructive sleep apnea, catecholamine, metanephrine

## Abstract

*Context*. As catecholamine elevation is a key element in the diagnosis of pheochromocytoma, more commonplace causes of sympathetic excess, such as obstructive sleep apnea (OSA), should be excluded as standard practice prior to diagnosis. This is essential to avoid misdiagnosis of adrenal incidentalomas identified in the estimated 42 million Americans with OSA, with greater than 4 million projected to undergo a computed tomography study annually. *Case Description*. A 56-year-old woman presented with a several year history of paroxysmal hypertension, palpitations, and diaphoresis. Abdominal/pelvic computed tomography performed during an unrelated hospitalization revealed a 2-cm left-sided adrenal nodule initially quantified at 37 Hounsfield units. Posthospitalization, 24-hour urine normetanephrine level was markedly elevated. Reassessment 2 weeks later revealed continued normetanephrine excess. Following normal thyroid function tests, morning cortisol, aldosterone, and plasma renin activity, laparoscopic adrenalectomy was performed. Surgical pathology identified an adrenal cortical adenoma. As paroxysms continued postoperatively, repeat 24-hour urine metanephrines were measured, demonstrating essentially unchanged normetanephrine elevation. Search for an alternate cause ensued, revealing OSA with progressive continuous positive airway pressure noncompliance over the preceding year. Regular continuous positive airway pressure therapy was resumed, and at the end of 7 weeks, 24-hour urine normetanephrine levels had declined. *Conclusion*. Pheochromocytomas are rare and sleep apnea is common. However, the overlap of clinical symptoms between these disorders is substantial, as is their ability to produce catecholamine excess. Thus, excluding uncontrolled or undiagnosed OSA in high-risk patients should be standard practice before diagnosing pheochromocytoma.

## Introduction

Obstructive sleep apnea (OSA) is a common disorder that can mimic not only the triad of diaphoresis, hypertension, and tachycardia associated with pheochromocytoma but also its biochemical profile of catecholamine excess.^1-3^ Adequate treatment with continuous positive airway pressure (CPAP) decreases both daytime and nighttime norepinephrine levels in patients with severe OSA. Conversely, withdrawal of CPAP therapy results in a rapid recurrence of urinary catecholamine elevation.^4^ Adrenal incidentalomas are frequently identified in patients undergoing imaging procedures, and measurement of plasma metanephrines or urinary metanephrines and catecholamines is recommended practice to rule out pheochromocytoma.^5^ Classic imaging characteristics of pheochromocytoma, such as increased Hounsfield units (HU) and low washout, can overlap with lipid-poor adenoma.^6^ Therefore, there is a reasonable likelihood of coincident confounders, leading to erroneous diagnosis of pheochromocytoma and unnecessary surgical intervention. While it is understood that workup for pheochromocytoma should not occur in acutely ill patients, more commonplace causes of catecholamine excess, such as OSA, also need to be excluded.

## Report of Case

A 56-year-old woman presented with a several year history of paroxysmal hypertension, palpitations, and diaphoresis without a clear inciting factor. A left sided adrenal incidentaloma had been recently discovered via an abdominal/pelvic computed tomography (CT) scan performed during a brief hospitalization for an unrelated issue. Follow-up 24-hour urine metanephrine levels performed several weeks following discharge demonstrated marked normetanephrine elevation of 2340 µg/24 hours, prompting referral for investigation of pheochromocytoma. Plasma normetanephrine was mildly elevated at 206 (normal is ≤148 pg/mL), but plasma metanephrine was within the reference range of ≤57 pg/mL. Medication review failed to identify anything that would lead to catecholamine excess, and she had no history of illicit drug use. Current medications included simvastatin, aspirin, metformin, and letrozole. A cigarette smoker for 30 years, tobacco use was unchanged in amount or frequency since symptom onset. Past medical history was notable for breast cancer in full remission, hypertension, nonproliferative diabetic retinopathy, ocular hypertension, and well-controlled type 2 diabetes mellitus. She had no history of psychiatric disorders, past or present. Family history included no endocrine disease, other than unspecified thyroid disorders, as well as coronary artery disease, stroke, and colon cancer.

Exam revealed an obese middle-aged woman. Body mass index was 43.8. Blood pressure was elevated at 166/100, with a pulse of 112. Obesity was mildly centrally predominant, without violaceous striae. Nonpitting edema was present in lower extremities without ecchymosis. The remainder of the physical exam was normal. Review of systems was positive for a documented 20-pound weight loss over the past 24 months, fatigue, chronic musculoskeletal pain, and heat intolerance. The abdominal/pelvic CT study was reviewed by an internal radiologist, who quantified the adrenal nodule HU at 37. Thyroid function tests (thyroid-stimulating hormone and free thyroxine), morning cortisol, aldosterone, and plasma renin activity were assessed and were within normal limits. Two weeks later, repeat 24-hour urine metanephrines demonstrated ongoing, albeit lesser elevation of normetanephrines at 900 µg/24 hours.

**Figure 1.**
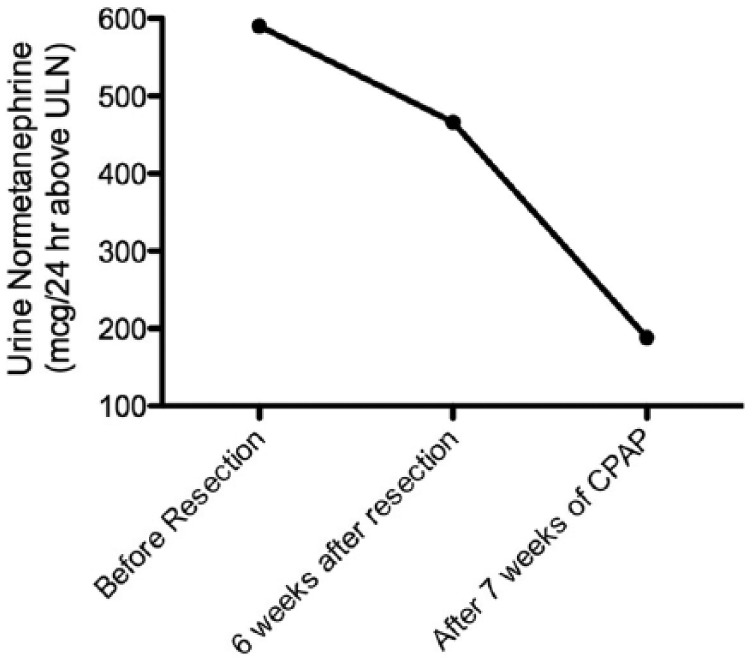
Urine normetanephrine levels, represented as g/24 hours greater than the upper limit of normal (ULN): prior to resection of adrenal gland, 6 weeks after adrenalectomy, and after 7 weeks of compliance with continuous positive airway pressure (CPAP) therapy.

Phenoxybenzamine was initiated, followed by β-blockade yielding substantial improvement in both paroxysmal hypertension and tachycardia in preparation for surgical resection. Laparoscopic adrenalectomy was performed for presumed pheochromocytoma. However, pathology revealed an adrenal cortical adenoma. The patient had continued paroxysms postoperatively, and a repeat 24-hour urine normetanephrine level remained elevated at 1142 µg/24 hours. We therefore looked for alternative underlying causes of sympathetic excess and discovered that the patient had progressive noncompliance with CPAP therapy for OSA over the 12 months prior to symptom onset. The patient was agreeable to resume regular CPAP use, and at the end of 7 weeks of CPAP compliance, repeat 24-hour urine normetanephrine levels had declined modestly. All the 24-hour urine metanephrine and catecholamine values obtained are listed in Table 1. Notably, the reference laboratory changed its urine metanephrine reference ranges substantially following this patient’s resection. Despite contacting the reference lab, we could not normalize the postoperative values to the preoperative values (details in Table 1). Therefore, we looked at the change in urinary normetanephrine over time by plotting the values as the absolute value (in µg/24 hours) above the upper limit of normal of the reference range (Figure 1).

**Table 1.** 24 hour urine metanephrine and catecholamine results over time.

Lab	9 Days s/p Hospitalization^a^	3 Weeks s/p Hospitalization^a^	6 Weeks s/p Adrenalectomy^b^	7 Weeks s/p CPAP Use^b^	Reference Range
Metanephrines					
Total	2512	978	1249	977	95-475 µg^a^; 224-832 µg^b^
Normetanephrine	2340	900	1142	864	52-310 µg^a^; 122-676 µg^b^
Metanephrine	172	78	107	113	19-140 µg^a^; 90-315 µg^b^
Catecholamines					
Norepinephrine	180	136	^c^	^c^	15-100 µg
Epinephrine	<10	<8	^c^	^c^	2-24 µg
Dopamine	304	231	^c^	^c^	52-480 µg

Abbreviation: CPAP, continuous positive airway pressure.

a Initial reference range.

b New reference range.

c Not tested.

## Discussion

The catecholamine excess resulting from untreated OSA is an important confounder in the biochemical assessment of adrenal incidentalomas or the workup for secondary causes of uncontrolled hypertension. OSA is a common sleep-related breathing disorder estimated to affect up to 30% of males and 15% of females in the United States.^7^ In patients with OSA, repetitive collapse of the upper airway results in hypopneic and/or apneic episodes. Patients with OSA commonly experience headache and hypertension, symptoms also suggestive of pheochromocytoma. Untreated OSA results in supraphysiologic levels of endogenous catecholamines.^1,3^ In addition to a general increase in sympathetic tone during waking hours, sympathetic activity increases markedly during nocturnal hypopneic and/or apneic episodes due to the stress of hypoxia, the jarring repetitive arousals, or a combination of all factors.^8-10^ Even in patients with well-controlled OSA, omission of CPAP therapy for even a few days has been shown to result in rapid resurgence of this sympathetic response.^4^ Although the association between untreated OSA and catecholamine elevation is well described in the area of sleep medicine and cardiology, there has only been one case series published in the endocrine literature.^3^ Thus, although other sources of false-positive catecholamine elevation such as medications and extreme illness are recognized in current guidelines for both the management of adrenal incidentalomas and diagnosis of pheochromocytoma, the impact of OSA, a chronic condition resulting in sympathetic excess, has not been addressed.^5,11^

Previous cases of OSA-related pseudopheochromocytoma have been reported.^3,12^ However, in the majority of these cases, clinical symptoms and catecholamine elevation were the factors inciting investigation. In our case, the discovery of an adrenal incidentaloma prompted the biochemical workup, revealing impressive elevations of normetanephrine levels in multiple 24-hour urine collections. While imaging studies may correctly identify a benign adenoma in some of these patients with untreated OSA, noncontrast HU measurements and CT washout characteristics of lipid-poor adenomas have a nontrivial amount of overlap with those of pheochromocytomas.^6^ In a retrospective review of our case, however, it was discovered that rather than a lipid poor adenoma, the HU were inadvertently reported using a contrast image.

Interestingly, our patient had a predominance of urinary normetanephrine, with minimal elevations in urine catecholamines. The adrenal medullary cells metabolize catecholamines to metanephrines, while catecholamines are released directly from sympathetic neurons. A predominance of metanephrine metabolite elevation versus catecholamine elevation is thought to suggest adrenal origin rather than excess sympathetic activity.^13^ Most of the literature on increased sympathetic activity in OSA has demonstrated catecholamine rather than metanephrine excess. Therefore, it is notable that our patient did not have significant urine catecholamine elevations. Also notable is the more dramatic elevation in urine normetanephrine (2.9-fold above normal), compared to plasma normetanephrine (1.4-fold above normal), in our patient prior to surgery. We can hypothesize that this may be related to the ability of the 24-hour urine collection to capture more excess sympathetic activity from overnight apnea events.

Plasma and urine metanephrines are both thought to deliver high sensitivity in detection of pheochromocytoma. There is some controversy with regard to differences in specificity, although recent Endocrine Society guidelines suggest that more false positives are seen in urine metanephrines.^11^ However, the reported specificity of either test ranges widely across studies, from 69% to 98%.^11,14^ There are no studies to inform us whether false positives from OSA-related sympathetic activity are more likely with plasma or urine metanephrines; however, in our case urine metanephrines were the most striking false positive. The earlier case series report describing similar “pseudopheochromocytoma” cases measured urine and plasma catecholamine levels (not metanephrines) and found roughly equivalent elevations in both plasma and urine samples.^3^

Similar to exclusion of other recognized sources of false-positive catecholamine and metanephrine elevation, untreated OSA should be considered as a legitimate confounder of biochemical analysis in testing for pheochromocytoma. Given the high prevalence of OSA and the growing number of adrenal incidentalomas identified in this era of increasing imaging, it is important to consider OSA as a reversible cause of sympathetic excess before making a diagnosis of pheochromocytoma. Thus, in cases in which the patient either has risk factors for OSA (ie, obesity,^15^ crowded pharyngeal airway,^16^ older age^17^) or questionable CPAP compliance in the setting of known OSA, we advocate that careful consideration is given to the possibility of a false-positive screening test result and OSA is first ruled out as a contributing factor prior to surgical resection.
